# Targeting Estrogen Receptor for Breast Cancer

**DOI:** 10.3390/cimb48070715

**Published:** 2026-07-13

**Authors:** Eugenia Yiannakopoulou

**Affiliations:** Department of Biomedical Sciences, Faculty of Health and Care Sciences, University of West Attica, 12243 Athens, Greece; nyiannak@uniwa.gr

**Keywords:** SERMS, SERDs, tamoxifen, fulvestrant, breast cancer, estrogen receptors, resistance, PROTACS

## Abstract

With a lifetime risk estimated to be 1 in 8 in industrialized countries, breast cancer is the most frequent type of cancer among women worldwide and the second leading cause of cancer deaths in women. More importantly, current evidence suggests that in women aged <45 years, breast cancer is unquestionably the leading cause of cancer-related deaths. Hormonal therapy has an established role in the treatment of breast cancer. Hormonal therapy aims at preventing the stimulation of mitogenic estrogen-dependent pathways. Hormonal therapy can be performed through blocking the production of estrogens or through blocking the action of estrogens upon tumor cells. The action of estrogens upon tumor cells can be blocked through selective estrogen receptor modulators (SERMs) or through selective estrogen receptor downregulators (SERDs). Estrogen receptor mutation (ESR1 mutation) is one of the common mechanisms by which breast cancer becomes resistant to additional therapies from SERMs or aromatase inhibitors. Fulvestrant, an injectable anti-estrogen, is the SERD commonly used. Fulvestrant has no agonistic activity and causes degradation of the estrogen receptor. This agent is more active in postmenopause than premenopause and is indicated in the treatment of advanced breast cancer in case of disease progression during or after tamoxifen. Oral SERDs are being rapidly developed to replace fulvestrant with the potential of higher efficacy and lower toxicities. Novel agents such as complete estrogen receptor antagonists (CERANs), proteolysis targeting chimeras (PROTACs), and selective estrogen receptor covalent antagonists (SERCAs) are also promising therapies. This manuscript focuses on recent advances in the development of drugs targeting the estrogen receptor.

## 1. Introduction

Breast cancer is the most common type of cancer among women worldwide and the second leading cause of cancer-related deaths among women. The lifetime risk of breast cancer is estimated to be 1 in 8 in industrialized countries [1]. More importantly, the literature data indicate that in young women aged <45 years, breast cancer is without any doubt the leading cause of cancer-related deaths. Breast cancer develops in epithelial cells of the mammary gland, with its development being highly influenced by a number of risk factors that involve prolonged exposure to estrogen and progesterone, i.e., early menarche, late menopause and an increasing number of productive hormonal cycles [2,3,4,5]. Estrogen and progesterone have a mitogenic effect in breast tissue and might lead to genomic instability and genetic alterations and contribute to the development of estrogen- and progesterone-positive breast cancer [6,7,8]. More than 70% of newly diagnosed breast carcinomas are recognized as hormone-sensitive breast cancers. These types of breast cancer are characterized by the expression of estrogen receptor and/or its transcriptional target progesterone receptor [9,10].

Hormonal therapy, including selective estrogen receptor modulators (SERMs), is the cornerstone of adjuvant therapy in patients with hormone-positive breast cancer. Hormonal therapy is also included in the treatment strategies employed in at least the first-line metastatic setting. Currently, there are three recognized classes of endocrine therapyfor hormone positive breast cancer. These classes include SERMs, aromatase inhibitors and selective estrogen receptor degraders (SERDs) [11]. SERMs are indicated in the adjuvant treatment of estrogen receptor-positive breast cancer in premenopausal and postmenopausal women, in chemoprevention for estrogen receptor-positive breast cancer as well as in metastatic estrogen receptor-positive breast cancer. SERMS comprise the first class of drugs of endocrine breast therapy, with tamoxifen being the prototype drug of this class. SERMs competitively inhibit the binding of estrogens to their receptors, thus preventing the estrogen receptor-initiated downstream signaling. These drugs present either agonist or antagonist activity, depending on the target tissue. While SERMs are effective at blocking the activation function 2 (AF2) domain, they are incapable of blocking the AF1 domain of ERα, which can lead to agonist activity and limit its effectiveness. Thus, although hormonal therapy is effective, it has the drawback of the development of acquired resistance to SERM treatment, which is manifested in clinical practice by the development of breast cancer disease progression in women treated with SERMS in the adjuvant setting.

The second class of endocrine therapy consists of the third-generation aromatase inhibitors. Aromatase inhibitors are indicated in the adjuvant treatment of estrogen receptor-positive breast cancer in postmenopausal women, in chemoprevention for estrogen receptor-positive breast cancer as well as in the treatment of metastatic estrogen receptor-positive breast cancer. These agents inhibit estradiol biosynthesis via aromatase inhibition, thus preventing estrogen receptor-mediated signaling. Anastrozole and letrozole are nonsteroidal aromatase inhibitors that noncovalently and reversibly competitively inhibit the binding of aromatase to androgens. On the other hand, exemestane is a steroidal aromatase inhibitor that covalently and irreversibly binds to the aromatase androgen-binding site.

The third class of breast endocrine therapy comprises the selective estrogen receptor degraders, including fulvestrant and elacestrant. The SERD elacestrant has received its first approval for the treatment of postmenopausal women or adult men with ER-positive, HER2-negative, estrogen receptor 1 (ESR1)-mutated advanced or metastatic breast cancer with disease progression following ≥1 line of endocrine therapy.

Targeting the cell cycle is a promising approach for estrogen receptor-positive breast cancer. In that sense, CDK4/6 inhibitors inhibit estrogen receptor downstream signaling from active estrogen receptors and thus can be administered in combination with endocrine therapy to overcome resistance to endocrine therapy. Currently, CDK4/6 inhibitors (palbociclib, ribociclib, abemaciclib) are often used in combination with aromatase inhibitors or selective estrogen receptor degraders in the adjuvant setting of advanced breast cancer or in the metastatic setting [12,13,14,15,16,17]. However, resistance to CDK4/6 inhibitors is also commonly encountered, raising the need for other approaches, such as targeting estrogen signaling downstream or upstream of estrogen receptors, i.e., use of PI3K/AKT/mTOR inhibitors in combination with endocrine therapy, maintenance of CDK4/6 inhibitors with endocrine therapy, and targeting other mediators of the cell cycle, i.e., CD2 and CD7 with endocrine therapy.

Overall, despite the effectiveness of endocrine therapy, the problem of the development of resistance to standard endocrine therapy and subsequent treatment failure remains a major limitation. In an effort to overcome resistance, novel drugs targeting the estrogen receptors directly have been developed.

This manuscript is a narrative review that critically presents recent advances in the development of endocrine therapy directly targeting the estrogen receptor for the adjuvant and metastatic treatment of estrogen receptor-positive breast cancer. Τhis review is structured in six sections, including the presentation of the role of estrogen receptors in the modulation of estrogen receptor-positive breast cancer, the main classes of endocrine therapy for estrogen receptor-positive breast cancer, selective estrogen receptor modulators, selective estrogen receptor modulator resistance and selective estrogen receptor degraders.

## 2. Estrogen Receptors in Modulation of Estrogen Receptor-Positive Breast Cancer

Estrogen receptors (ERs), namely ERα and ERβ, are members of the nuclear receptor superfamily whose members share conserved domain structures, including a DNA-binding domain and ligand-binding domain. Both ERs share a conserved nuclear receptor domain structure that encompasses a DNA-binding domain, ligand-binding domain (LBD), a central hinge region, and two activation functional domains (AFs). The transcriptional activity is dependent on the two activation functional domains. The AF-1 operates in a ligand-independent mode, while AF-2 is ligand-dependent. The ligand binding to ERα or ERβ induces a conformational change that leads to receptor dimerization, leading to the formation of homodimers (ERα/α or ERβ/β) or heterodimers (ERα/β). The ERα/α homodimer promotes, and the ERβ/β inhibits estrogen-dependent growth of mammary epithelial cells. The functions of ERα/β heterodimers remain under investigation.

When the ERα–ligand complex binds to genomic sequences, often called estrogen receptor elements (ERE), it interacts with coregulators in order to modulate the transcription of target genes.

ERα is the predominant receptor isoform expressed in breast cancer cells, and approximately 70% of breast cancer patients score positive for ERα upon diagnosis. Tamoxifen and the other selective estrogen receptor modulators act via modulation of estrogen receptors. Thus, understanding the biology of estrogen receptors is critical for understanding how resistance to selective estrogen receptor modulators develops.

Multiple mechanisms modulate the oncogenic functions of ERα in breast cancer. ERα mediates gene expression via a process that is highly regulated by complex interactions with coactivators and corepressors. In that process, the role of coactivators is the potentiation of ERα-dependent transcription via the recruitment of histone acetyltransferases. On the other hand, ERα corepressors inhibit ERα-mediated gene transcription via recruitment of histone deacetylases, via competing with coactivators for the same binding sites on transcription factors and via interference with ERα dimerization. Data using microarray analysis from MCF cancer cell lines suggest that the majority of estrogen-regulated genes are downregulated by treatment with estradiol [18]. Estrogen receptor-mediated transcription is further mediated by co-regulatory proteins including corepressors and coactivators. Co-activators enhance transcription, while corepressors suppress transcription [19,20,21,22,23,24]. The co-activators associate with each other as well as with the general transcription machinery of the cell, thus forming large complexes that are capable of synergistically activating estrogen-driven transcription.

The antineoplastic mechanism of action of tamoxifen in breast cancer is based on tits competition with estrogen for the ligand-binding domain of estrogen receptor alpha. Thus tamoxifen induces a conformational change that enhances ERα’s interaction with transcriptional corepressors. The net effect is that tamoxifen achieves its antineoplastic action by repressing ERα-mediated transcription, inducing cell cycle arrest and leading to cell death.

De novo resistance to tamoxifen treatment derives primarily from loss of ERα expression. However, the biological mechanism underlying acquired endocrine resistance is not adequately understood. It is known that patients with tumor progression while on adjuvant treatment with tamoxifen continue to express estrogen receptors in tumor cells, suggesting that mechanisms other than loss of ER receptor signaling have been associated with resistance to endocrine therapy. Thus, existing data suggest that the acquisition of endocrine resistance is often coincident with a shift from ligand-dependent to ligand-independent control of ERα-regulated breast cancer cell growth and survival, possibly due to bidirectional molecular crosstalk between ERα and growth factor signaling pathways. Thus, tamoxifen resistance could be partly attributed to ligand-independent activation of ERα. In an effort to further elucidate this path, researchers have undertaken proteomics-based approaches to isolate ligand-independent ERα networks.

## 3. Endocrine Therapy for Estrogen Receptor Positive Breast Cancer

Hormonal therapy has an established role in the treatment of breast cancer. Hormonal therapy aims at preventing the stimulation of mitogenic estrogen-dependent pathways. Hormonal therapy can be performed through blocking the production of estrogens or through blocking the action of estrogens upon tumor cells [Table 1].

i. Medical Inhibition of Ovarian Function

Estrogen production can be blocked through ovarian inhibition that could be surgical through oophorectomy or radiological or medical. Medical inhibition of ovarian function can be performed through the administration of LH-RH analogs or through the administration of aromatase inhibitors, which inhibit aromatase, an enzyme that converts androgen precursors to estrogens. LH-RH analogs, including goserelin, triptoreline, and buserelin, produce, in a reversible manner, a reduction in the circulating concentrations of estrogen (ovarian suppression) in premenopausal women through their action on the hypothalamus–pituitary–ovarian axis. This is due to the fact that continuous administration of GnRH agonists produces early receptor activation and subsequent downregulation and desensitization in gonadotropic cells. These effects decrease the secretion of LH and FSH and lead to a lack of sex-steroid hormones, including estrogens, testosterone, and progesterone, with the concomitant appearance of “chemical castration”.

According to current guidelines, GnRH analogs are indicated in combination with tamoxifen or aromatase inhibitors for the adjuvant therapy of premenopausal women with estrogen receptor-positive breast cancer (often preceded by chemotherapy). This strategy is due to the results of The Tamoxifen and Exemestane Trial (TEXT) and the Suppression of Ovarian Function Trial (SOFT), which have shown the superior efficacy of exemestane or tamoxifen in combination with the suppression of ovarian function versus tamoxifen alone or aromatase inhibitors alone [25,26,27,28,29,30]. However, concerns of suboptimal estrogen suppression in premenopausal women treated with GnRH agonists and aromatase inhibitors have been expressed, and suggestions of estrogen level measurements during GnRH agonist–aromatase inhibitor therapy have been proposed.

ii. Inhibition of estrogen biosynthesis

Aromatase inhibitors are well established in the treatment of postmenopausal women with hormone-positive breast cancer [31]. Although after menopause, estrogens are no longer produced by the ovaries, peripheral tissues produce sufficient concentrations to stimulate tumor growth. Aromatase inhibits the final and rate-limited step in the biosynthesis of estrogens. Aromatase inhibitors currently used are third-generation agents, including the reversible non-steroidal aromatase inhibitors anastrozol at the dosage of 1 mg/day p.o and letrozol at the dosage of 2.5 mg/day p.o or the irreversible steroidal exemestan at the dosage of 25 mg/day p.o. Aromatase inhibitors are indicated only in postmenopausal patients as adjuvant as well as first-line metastatic treatment. No clinically significant difference has been evidenced among the three aromatase inhibitors, although letrozole has been reported to result in higher levels of aromatase inhibition based on blood measurements.

iii. Directly Targeting the Estrogen Receptor

The action of estrogens upon tumor cells can be blocked through SERMs or through selective estrogen receptor downregulators (SERDs).

Although SERMS and aromatase inhibitors are efficient endocrine therapies for estrogen receptor positive breast cancer, breast cancer cells can develop resistance to these therapies with estrogen receptor mutation (ESR1 mutation) being one of the important mechanisms that lead to the appearance of acquired resistance. The implicated ESR1 mutations are located in the ligand-binding domain of the receptor and lead to estrogen independent constitutive estrogen receptor activation. Apart from mutations, ESR1 alterations, including amplifications, are identified in up to 30% of women with estrogen receptor positive breast cancer. However, the clinical significance of these alterations has not been yet fully elucidated. Furthermore, ESR1 fusions need further investigation, as at the moment their clinical implications are not known.

Fulvestrant, an injectable anti-estrogen, is the SERD most commonly used. Fulvestrant has no agonistic activity and causes degradation of estrogen receptor [32]. This agent is more active in postmenopause than premenopause and is indicated in the treatment of advanced breast cancer in case of disease progression during or after tamoxifen [33]. Currently, oral SERDs have been developed and are under comparison with fulvestrant in a number of clinical trials, in terms of efficacy and tolerability [34]. Elacestrant is the first oral SERD that was investigated in a phase III randomized clinical trial and demonstrated promising results in terms of efficacy and tolerability, especially for patients with breast cancer tumors bearing ESR1 mutation.

iv. Novel approaches directly targeting the estrogen receptor

Novel agents such as complete estrogen receptor antagonists (CERANs), proteolysis targeting chimeras (PROTACs), and selective estrogen receptor covalent antagonists (SERCAs), selective human ER partial agonists (ShERPAs) are also promising therapies [35,36,37,38,39,40,41] [Table 1].

CERANS block entirely estrogen receptor expression pathway by blocking both transcriptional activation domains of estrogen receptor. The estrogen receptor includes two distinct transcriptional activation domains i.e., transcriptional activation domain AF1, that is activated by signaling pathways such as mTOR, PI3K, and MAPK and transcriptional activation domain AF2, that is activated by the estrogen ligand itself. Palazestrant (OP-1250) is an orally bioavailable agent that functions as both complete estrogen receptor antagonist and selective estrogen receptor degrader that has entered clinical development [42]. Palazestrant is under investigation in patients with advanced or metastatic estrogen receptor positive breast cancer [43] Based on the promising results of Phase I/II trials, palazestrant has recently entered a Phase III trial (OPERA I).

Selective estrogen receptor covalent antagonists (SERCAs) inactivate both ER wild-type and mutated ER by covalently targeting a unique cysteine residue [cysteine-530 (C530)] that is not present in other hormone receptors. The orally bioavailable SERCA, H3B-6545, has shown antitumor activity in heavily pretreated patients with ER+ metastatic breast cancer as a single agent. H3B-6545 is currently being evaluated in phase I and II settings as a monotherapy (NCT03250676) and in combination with palbociclib (NCT04288089) [44]. Adverse events including asymptomatic sinus bradycardia and QTc prolongation have been reported with H3B-6545.

PROTACs are bifunctional hybrids that simultaneously bind to a specific target protein, such as ER, and an E3 ubiquitin ligase, resulting in ubiquitination and degradation of the target protein ER through the ubiquitin-proteasome system. The ubiquitin-proteasome system is a highly conserved mechanism for degradation of both normal and misfolded proteins in eukaryotic cells, thus maintaining intracellular protein homeostasis. PROTACs can degrade almost all intracellular proteins, including protein kinases, nuclear receptors, and transcriptional factors. Cell surface proteins are not considered optimal targets for PROTACs. The mechanism of action of PROTACs is catalytic, enabling them to enhance protein degradation even at low concentration levels.

Computer simulation technology has importantly accelerated PROTAC design. The first-generation PROTACs, due to their peptidic nature, exhibited low cell permeability and stability in biological systems, a characteristic that greatly limited their further development. At first, a polycationic polyarginine chain was added to the first-generation PROTAC structure, in an attempt to improve the cell permeability issue. However, the real progress in the field came with the development of small-molecule-based PROTACs, which could be further improved to drug-like molecules. What is really promising with PROTACs is that these molecules share a unique advantage over conventional therapeutic approaches, including small-molecule inhibitors and monoclonal antibodies. PROTACs function via the “event-driven” mechanism, which allows them to exert their effect even in low concentrations.

PROTACs are promising strategies against breast cancer. Importantly, the first PROTAC has been recently approved for estrogen-positive breast cancer. The oral PROTAC estrogen receptor degrader Vepdegestrant (ARV-471) has been investigated versus fulvestrant in a Phase III clinical trial in patients with advanced ER+/HER2- breast cancer previously treated with a CDK4/6 inhibitor and endocrine therapy. In a previous phase I/II study, vepdegestrant monotherapy was well tolerated and demonstrated clinical efficacy in previously treated women with advanced ER+/HER2- breast cancer. Recently, on 1st May 2026, the FDA approved vepdegestrant for the treatment of patients with ER-positive HER2-negative ESR1-mutated advanced or metastatic breast cancer who present with disease progression after at least one line of endocrine therapy.

In addition, novel promising protein degradation technologies have been developed, such as molecular glue degrader (MGD) and lysosome-targeting chimeras (LYTACS). These technologies are expected to be efficient ER degraders with possible clinical applications. In the meantime, ribonuclease-targeting chimeras (RIBOTAC) and small interfering RNA (siRNA) are promising strategies for the inhibition of the production of ER proteins.

Targeted protein degradation is a therapeutic approach that aims to selectively remove disease-causing or undesirable proteins from cells by inducing their degradation, with multiple therapies entering clinical trials and targeting proteins that are previously considered “undruggable”. Targeted protein degradation with molecular glue degraders has arisen as a powerful therapeutic modality for eliminating classically undruggable disease-causing proteins through proteasome-mediated degradation. Molecular glue degraders are molecules that encourage two proteins to come together that normally would not interact. These molecules achieve this by changing the surface of their target proteins. LYTACS induce selective degradation of extracellular proteins by recruiting them to cellular receptors that mediate delivery to the lysosome. The first LYTAC molecule was published in *Nature* in 2020. While most drugs target proteins, several diseases are associated with the presence or absence of several RNAs, so targeting RNA could also be a therapeutic approach.

## 4. Selective Estrogen Receptor Modulators

Selective estrogen receptor modulators (SERMs) are synthetic non-steroidal agents that have varying estrogen agonist and antagonist activities in different tissues, most likely due to the receptor conformation changes associated with the mode of SERMs’ binding and the subsequent effect on transcription [45]. SERMs can be classified based on their chemical structure as triphenylethylenes (tamoxifen, 4-hydroxytamoxifen, endoxifen, toremifene, droloxifene, idoxifene), benzothiophenes (raloxifene, arzoxifene), phenylindoles (bazedoxifene, pipindoxifene), and tetrahydronaphthalenes (lasofoxifene). All selective estrogen receptor modulators are characterized by a core scaffold that mimics the chemical structure of 17β-estradiol and confers high binding affinity for estrogen receptors.

Tamoxifen is the SERM most commonly used in the targeted treatment of hormone positive breast cancer. The standard approach was treatment with tamoxifen for five years in the adjuvant setting. However, currently the extended adjuvant tamoxifen therapy is strongly proposed based on the results of trials of 5 versus 10 years of adjuvant tamoxifen therapy that have demonstrated the superiority of adjuvant tamoxifen treatment for 10 years versus the standard 5 year duration. The ATLAS (Adjuvant Tamoxifen Longer against Shorter) trial included breast cancer patients who had been treated with 5 years of tamoxifen in the adjuvant setting. Patients were randomly assigned into two groups: one group would continue receiving tamoxifen for an overall duration of 10 years and the other group would stop treatment. According to the results of the trial, allocation to adjuvant tamoxifen therapy for an overall duration of 10 years resulted in reduction of the risk of breast cancer recurrence, reduction of breast cancer mortality and overall mortality. In accordance with ATLAS, aTTom Study showed that extended tamoxifen use resulted in lower the incidence of breast cancer recurrence as well as lower breast cancer-related mortality. However, treatment prolongation results in the increase of endometrial cancer as well as in the increase of thromboembolic events and the development of acquired resistance [46,47]. 

It is well established that adjuvant endocrine therapy with tamoxifen or aromatase inhibitors, reduces recurrence risk and increases survival among patients with hormone receptor positive breast cancer [48]. According to the National Comprehensive Cancer Network (NCCN) guidelines the following options are recommended for the adjuvant treatment of postmenopausal estrogen receptor positive breast cancer patients: (1) tamoxifen for a duration of 5 to 10 years, (2) an aromatase inhibitor for a duration of 5 to 10 years, (3) tamoxifen for 5 years, followed by an aromatase inhibitor for 5 years, (4) tamoxifen for 2–3 years, followed by an aromatase inhibitor for 5 years, or (5) an aromatase inhibitor for 5 years followed by tamoxifen for 5 years. 

The limitation of adjuvant endocrine therapy is the development of resistance in a number of women who initially respond who will eventually develop disease progression and relapse while on therapy. In estrogen receptor positive breast cancer, it is estimated that 40–50% of breast cancer patients treated with tamoxifen will eventually develop tamoxifen resistance and relapse [49,50,51,52]. Importantly the five year survival rate following tamoxifen resistance is less than 20% [53,54,55,56,57].

Raloxifene, a second-generation SERM, is a benzothiophene derivative that was first approved for the treatment of postmenopausal osteoporosis. Furthermore, raloxifene has a significant impact on the prevention of breast cancer [57]. Subsequently, research efforts continued with the aim of obtaining compounds with a better activity and safety profile, leading to a third generation of SERMs characterized by improved potency. Computational chemistry has contributed to the design of third-generation SERMs. An interesting molecular docking study utilized Raloxifene as one of the compounds to design modafinil drug derivatives as inhibitors for estrogen receptor alpha (ER*α*) of breast cancer [58].

Arzoxifene was a benzothiophene analog of raloxifene that had been investigated in tamoxifen-resistant patients. A phase III trial had been designed to compare arzoxifene with tamoxifen; however, the trial was terminated as, in the interim analysis, arzoxifene was found to be inferior to tamoxifen with respect to the time to progression interval.

Lasofoxifene, a third-generation SERM, is a naphthalene derivative, currently under investigation in patients with advanced estrogen receptor-positive breast cancer and ESR1 mutations [59]. Lasofoxifene had been initially developed for the treatment of osteoporosis, and in that context, investigators found that it reduced breast cancer incidence. Experimental data from relevant mouse models suggest that lasofoxifene might be promising for the treatment of aromatase inhibitor-resistant estrogen receptor-positive breast cancer independent of the presence of ESR1 mutations [60]. Elaine 1, an open-label randomized phase 2 study, was the first trial that compared lasofoxifene with the estrogen receptor degrader fulvestrant in patients with locally advanced or metastatic estrogen receptor-positive breast cancer and the presence of ESR1 mutations, previously treated with CDK 4/6 inhibitors [58]. Elaine 2 is a phase 2 single-arm study that evaluates the toxicity and efficacy of lasofoxifene with abemaciclib in women with metastatic ER+/HER2− breast cancer and an ESR1 mutation previously pretreated [61]. Regarding the safety of lasofoxifene, there is knowledge from fracture prevention studies, as lasofoxifene had been approved for the treatment of osteoporosis in some European countries, but the approval was withdrawn in 2012. Treatment with lasofoxifene for more than 5 years has been shown to be associated with benign endometrial hypertrophy [62].

Bazedoxifene is a third-generation SERM. Bazedoxifene binds to both ERα and ERβ, with a slightly higher affinity for ERα; however, it is less ERα-selective than raloxifene, with an affinity for ERα that is approximately 10-fold lower than 17β-estradiol (E2). Bazedoxifene is approved in Europe and is under regulatory review in the United States for the prevention and treatment of postmenopausal osteoporosis. However, in some contexts, bazedoxifene displays a “SERD-like” profile, and therefore it is often referred to as a mixed SERM/SERD hybrid. Bazedoxifene is effective in reducing cancer progression through multiple mechanisms. Bazedoxifene could effectively inhibit STAT3, PI3K/AKT, and MAPK signaling pathways and induce apoptosis. In addition to its anticancer activity as monotherapy, bazedoxifene has been shown to enhance the chemotherapeutic efficacy of clinical drugs such as paclitaxel, cisplatin, palbociclib, and oxaliplatin in multiple neoplasms. In breast cancer, bazedoxifene acts as a potent antagonist inhibiting E2-induced proliferation in MCF7 cells. Experimental data show that bazedoxifene is effective in tamoxifen-resistant breast cancer cells as well as in patient-derived xenograft (PDX) models [63,64]. Bazedoxifen has been approved for the treatment of postmenopausal symptoms and osteoporosis. In addition, bazedoxifene is currently being investigated in combination with palbociclib in women with advanced ER+ breast cancer.

Pipindoxifene is another selective estrogen receptor modulator that has been shown to inhibit estrogen-mediated growth of breast cancer cells and has also shown efficacy in tamoxifen-resistant tumors. While it was well tolerated in the clinical setting and a phase II study had begun for the treatment of tamoxifen-resistant metastatic breast cancer, it was later discontinued in 2005 because pipindoxifene had been developed as a backup agent for bazedoxifene. Since bazedoxifene proceeded to further development, pipindoxifene was discontinued.

## 5. Selective Estrogen Receptor Modulator Resistance

There are three types of resistance to SERMs: metabolic resistance, de novo resistance and acquired resistance [65,66]. Metabolic resistance is due to pharmacogenomic factors affecting the metabolism of the prodrug tamoxifen to its active metabolites, i.e., 4-hydroxytamoxifen and endoxifen that drive the therapeutic potential of tamoxifen. CYP2D6 is a major enzyme involved in tamoxifen metabolism, with its polymorphisms frequently leading to resistance to tamoxifen therapy [67]. Acquired resistance affects the long-term inhibition of breast cancer growth. The acquired resistance to SERMS is quite impressive in the sense that while at first SERMs inhibit estrogen action in breast cancer cells and inhibit breast cancer growth, they subsequently cause SERM-stimulated breast cancer growth, meaning that after years of treatment, cell populations that are adapted to grow in an antiestrogenic environment now dominate.

Different mechanisms have been implicated in the process of acquisition of estrogen treatment resistance. Implicated mechanisms include amplification of Her2; activation of alternative signaling pathways such as increased mitogen-activated protein kinases (MAPK) signaling; alterations in the direct targets of tamoxifen, such as shift from ligand-dependent to ligand-independent control of ERα or downregulation of ERα corepressors; alterations in cytochrome (CYP) gene expression; altered expression of specific microRNAs; autophagy; and genetic polymorphisms involved in tamoxifen metabolic activity [68,69,70,71,72,73,74,75,76,77]. The thorough investigation of these mechanisms is quite important, as modulation of these mechanisms could lead to strategies that would effectively circumvent SERM resistance [78,79,80,81].

Moreover, the identification of biomarkers and diagnostics is needed that could allow the early identification of breast cancer patients who could fail SERM treatment. In that context, more than 60 different mitochondrial markers have been identified that can be used individually or in combination, as short signatures, to predict tumor recurrence in tamoxifen-treated breast cancer patients. Furthermore, it has been suggested that mitochondria could be a potential pharmacological target for overcoming resistance to hormonal therapy and preventing tumor recurrence and distant metastasis [82]. In that aspect, metformin, a mitochondrial complex I inhibitor, has been shown to overcome tamoxifen resistance in estrogen receptor-positive cell culture models, which mimic the tumor microenvironment by the addition of stromal fibroblasts [83,84,85,86]. Apart from inhibiting mitochondrial complex I, metformin has been shown to inhibit the expression and function of ERα [83]. However, further research on the potential effect of metformin in the reversal of tamoxifen resistance is needed, as there are experimental data that suggest the development of cross-resistance to metformin and tamoxifen in breast cancer cells. Importantly, the acquired resistance to both drugs is based on the constitutive activation of Akt/Snail1/E-cadherin signaling, a finding that can also be exploited in the identification of future drug targets for overcoming tamoxifen resistance.

## 6. Selective Estrogen Receptor Degraders

Selective estrogen receptor degraders (SERDs) bind to estrogen receptors and promote its degradation. This mechanism of action of SERDs renders them highly promisive in overcoming estrogen receptor positive breast cancer acquired resistance, as SERDs function not only as competitive estrogen receptor antagonists, but also induce proteasome-dependent degradation of estrogen receptor. The role of estrogen receptor itself is crucial in the development of estrogen receptor positive breast cancer acquired resistance in endocrine therapy. It is well known that acquired resistance in ER-positive breast cancer is commonly developed from ligand-independent activation either through direct mutation of estrogen receptor or phosphorylation of estrogen receptor or its coregulators through signaling pathways such as PI3K-AKT-mTOR. 

Fulvestrant (Faslodex) was introduced as endocrine therapy of estrogen receptor positive HER2-negative breast cancer in 2002. Fulvestrant has been shown to be effective as both first- and second-line therapy for metastatic estrogen receptor positive breast cancer [87,88,89,90]. In addition, it has been demonstrated that fulvestrant has increased efficacy in combination with cyclin-dependent kinase (CDK) 4/6 inhibitors such as palbociclib [88,89], and abemaciclib (MONARCH 2 trial) [13] as the second-line therapy and ribociclib (MONALEESA-3) [15,90] as the first- or second-line therapy in metastatic estrogen receptor positive breast cancer compared to fulvestrant alone. 

Fulvestrant is injected intramuscularly. Currently, there are ongoing research efforts into the development of a new generation of SERDs that have excellent bioavailability and can be administered orally. Numerous oral SERDs are in clinical development, including giredestrant, amcenestrant, camizestrant, elacestrant, and rintodestrant. Importantly, some of the oral SERDs have shown efficacy in ESR1-mutated breast cancer cell lines and PDX models that have shown complete resistance to fulvestrant [91,92]. Initial clinical trial data have demonstrated that tumors without the ESR1 mutation are less likely to benefit from the SERDs and may still respond to SERMs or aromatase inhibitors, including tumors previously exposed to hormonal therapy [93].

Elacestrant is an orally available SERD for the treatment of estrogen receptor (ER)-positive, human epidermal growth factor receptor 2 (HER2)-negative breast cancer. Elacestrant inhibits gene transcription, induction, and cell proliferation, specifically in ER+ breast cancer cell lines [94,95,96,97,98]. Molecular docking studies have investigated the interaction of elacestrant with the estrogen receptor. Elacestrant binds with the helices H3, H5, H6, and H11 and forms important intermolecular interactions [99]. In January 2023, elacestrant received its first approval for the treatment of postmenopausal women or adult men with ER-positive, HER2-negative, estrogen receptor 1 (ESR1)-mutated (as determined by a US FDA-approved test) advanced or metastatic breast cancer with disease progression following ≥1 line of endocrine therapy in the USA. Elacestrant is a dose-dependent mixed ER agonist/antagonist, which at high doses acts as a direct ER antagonist as well as a selective downregulator of ER.

In a phase-1 study RAD1901-005 (ClinicalTrials.gov ID: NCT02338349), patients with advanced ER+ breast cancer were enrolled in dose escalation cohorts, followed by a safety expansion cohort. Key inclusion criteria include postmenopausal women aged 18 years or older, with advanced ER+, HER2- breast cancer, who have received ≤2 prior chemotherapy regimens in the metastatic setting and > 6 months of prior endocrine therapy. Elacestrant demonstrated evidence of single-agent activity, with confirmed partial responses in heavily pre-treated patients with advanced ER+ breast cancer, including those with ESR1 mutations, warranting additional clinical development.

Current clinical trials are ongoing, evaluating it in the adjuvant setting in patients with early-stage ER-positive breast cancers as well as in patients with advanced breast cancer [100,101]. EMERALD is a phase III randomized open-label clinical trial that evaluated the efficacy and safety of elacestrant compared with standard-of-care endocrine therapy in patients with ER-positive/HER2-negative advanced or metastatic breast cancer who had progression after first- or second-line treatment with the combination of endocrine therapy and a CDK4/6 inhibitor. The study also compared efficacy between arms in patients with detectable ESR1 mutation. Standard of care endocrine treatment included fulvestrant, anastrozole, letrozole, or exemestane monotherapy according to the investigator’s chοice. Patients could also have previously taken one chemotherapy regimen. The primary end points were progression free survival (PFS) in all patients and in patients with detectable ESR1 mutation. Elacestrant demonstrated a significant PFS improvement versus standard of care endocrine therapy both in the overall population and in patients with ESR1 mutations [102]. The most common adverse effects observed with elacestrant included nausea (35.0% vs. 18.8% in the standard of care endocrine treatment arm), fatigue (19.0% vs. 18.8% in the standard of care endocrine treatment arm), vomiting (19.0% vs. 8.3% in the standard of care endocrine treatment arm), decreased appetite (14.8% vs. 9.2% in the standard of care endocrine treatment arm), and arthralgia (14.3% vs. 16.2% in the standard of care endocrine treatment arm). Grade 3/4 adverse events occurred in 64 patients (27.0%) receiving elacestrant and 47 patients (20.5%) receiving standard of care endocrine therapy.

Elacestrant is available in tables of 345 mg and 85 mg. The standard dose of elacestrant is 345 mg, administered orally, once a day until disease progression or appearance of unacceptable toxicity. Therapy with elacestrant is not recommended in patients receiving strong or moderate cytochrome P450 (CYP)3A4 inducers and inhibitors and in those with severe hepatic impairment (Child-Pugh C). 

Amcenestrant (SAR439859) is another oral SERD [91,92,93,94] that has been investigated in preclinical and clinical studies [103,104,105]. Amcenestrant has been investigated in multiple estrogen receptor positive breast cancer cell lines, including fulvestrant- and tamoxifen-resistant lines, as well as in breast cancer cell lines harboring ER mutations [103]. In vitro studies have shown that amcenestrant antagonizes the binding of estrogen to estrogen receptor. In addition in vitro data have shown that amcenestrant promotes the transition of ERα to an inactive conformation. 

The clinical effect of amcenestrant has been evaluated in clinical phase I/II trials [104,105]. However the development of amcenestrant was discontinued in 2022 as the agent failed to meet the boundaries for continuation in interim phase analysis of the corresponding clinical trials. 

The next generation oral SERD Camizestrant is a pure ER antagonist. Camizestrant has been thoroughly investigated in estrogen receptor positive breast cancer cell lines. The in vitro experimental data have shown that camizestrant degradates ERa receptor to the same extent as fulvestrant and completely antagonizes gene expression induced by estradiol [106,107,108]. Importantly, camizestrant given as monotherapy as well as when administered in combination with CDK4/6 inhibitors has demonstrated antitumor effects in experimental models of ER+ breast cancer that bear clinically relevant mutations in ESR1 [109]. 

SERENA 1 was a phase I, open-label, dose-dependent exposure trial that analyzed the safety, tolerability, and preliminary clinical efficacy of camizestrant monotherapy, and in combination with palbociclib, everolimus, abemaciclib, and capivasertib in pretreated women with ER+ HER2− advanced breast cancer [110]. As monotherapy, camizestrant at all doses demonstrated an objective response rate of 15.3 %, clinical benefit rate at 24 weeks of 35.2 % and a PFS of 5.4 months. The majority of treatment-related adverse effects were of grade 1 or 2 severity and commonly included anaemia, fatigue, lymphopenia, nausea, neutropenia, thrombocytopenia, and reduced white blood cell count. SERENA 2 was a Phase 2 randomized open label study that compared the efficacy of camizestrant versus fulvestrant in women with estrogen receptor positive HER2– advanced breast cancer [111]. In this study camizestrant at doses of 75 and 150 mg achieved a statistically significant better median progression free survival in comparison with fulvestrant. SERENA-4 was a phase 3 clinical trial that compared camizestrant and palbociclib versus anastrozole and palbociclib as first-line treatment for women with estrogen receptor positive /HER2– advanced breast cancer [112]. SERENA-6 (NCT04964934) is a randomized controlled double blind phase III clinical trial that compared the effects of switching to camizestrant in combination with palbociclib or abemaciclib versus continuing anastrozole or letrozole in combination with palbociclib or abemaciclib in patients with ER+/HER2− metastatic breast cancer with detectable ESR1m, who are already receiving first-line treatment [113]. Based on the results of SERENA-6, camizestrant in combination with a CDK4/6 inhibitor has already been approved in the United Arab Emirates and Saudi Arabia for the treatment of women with estrogen receptor positive HER2 negative advanced breast cancer with detectable ESR1 mutations. Furthermore, based on the results of SERENA-6, camizestrant in combination with a CDK4/6 inhibitor (palbociclib, ribociclib, or abemaciclib) is under evaluation by FDA and EMA. A positive opinion has been granted by the Committee for Medicinal Products for Human Use (CHMP) on 21 May 2026. The most common adverse events of camizestrant combined with a CDK 4/6 inhibitor include neutropenia, visual effects, infections, anaemia, diarrhoea, nausea, fatigue, bradycardia and leukopenia.

Rintodestrant is another example of an orally bioavailable SERD. Treatment with rintodestrant has shown inhibition of estrogen-mediated transcription and proliferation in ER+ breast cancer cells, that are similar to the effects of fulvestrant. In addition, rintodestrant can effectively suppress ER activity in multiple experimental breast cancer models of endocrine resistance including models harbouring ESR1 mutations as well as activation of growth factors. In addition to its direct antagonist properties, the mechanism of action of rintodestrant includes reducing levels of estrogen receptor protein through proteasome-mediated degradation. Rintodestrant is currently evaluated in phase I clinical trial as a monotherapy and combined with Palbociclib [114]. These data have shown that rintodestrant could potentially be promising in the treatment of ER+ breast cancer either alone or in combination with CDK4/6 inhibitors. 

Imlunestrant is a next-generation oral SERD that penetrates the brain [115,116]. Since September 2025, imlunestrant has been approved by the FDA for the treatment of ER-positive, HER2-negative, ESR1-mutated advanced or metastatic breast cancer that has progressed after at least one line of endocrine therapy. EMBER was a phase 1a/1b study that investigated the effect of imlunestrant administered at doses ranging from 200 mg to 1200 mg. In the Phase Ia study, imlunestrant was given as a monotherapy in patients with advanced ER+ HER2− breast cancer that have been treated with three or fewer therapies previously [109]. In the Phase Ib study, imlunestrant was given in combination with abemaciclib +/− an aromatase inhibitor (anastrozole, exemestane, or letrozole) [117,118,119,120]. Imlunestrant administered at a dose of 400 mg daily exhibited an acceptable safety profile, with most documented adverse events being low-grade, reversible gastrointestinal symptoms [118]. The phase III EMBER-3 trial is evaluating the effectiveness of imlunestrant with or without abemaciclib in comparison to fulvestrant or exemestane, for the treatment of hormone-sensitive HER2-negative advanced breast cancer following progression on aromatase inhibitors. In that trial, primary end points were investigator-assessed progression-free survival with imlunestrant as compared with standard therapy among patients with ESR1 mutations and among all patients, as well as progression-free survival with imlunestrant–abemaciclib as compared with imlunestrant among all patients who had undergone randomization concurrently. EMBER 3 study results indicated that treatment with imlunestrant led to significantly longer progression-free survival than standard therapy among those with ESR1 mutations but not in the overall population. Furthermore, according to the data, treatment with the combination of imlunestrant with abemaciclib led to a significantly improved progression-free survival in comparison with imlunestrant monotherapy in breast cancer patients with ESR1 wild type as well as in patients with mutated ESR1 [115,121]. EMBER-4 is another phase 3 study that evaluates imlunestrant compared to exemestane or fulvestrant in patients suffering from high-risk ER+ HER2− early breast cancer [122]. Patients should have been treated with two to five years of adjuvant endocrine therapy prior to randomization [122]. Furthermore, imlunestrant is evaluated in the neoadjuvant setting.

Giredestrant, another orally available SERD, is highly potent, competes against E2 for binding, and drives an antagonist conformation within the estrogen receptor ligand-binding domain, thus inducing ER turnover and suppressing ER transcriptional activity, resulting in a robust antiproliferative effect. Giredestrant is commonly evaluated in combination with CDK4/6 inhibitors in the treatment of advanced HR-positive breast cancer [123,124,125]. Importantly, giredestrant has been also evaluated in early estrogen receptor positive breast cancer.

GDC-0810 (brilanestrant) is a non-steroidal oral SERD that exhibits nM range of binding affinity and efficacy. Molecular docking analysis studies have investigated the binding nature of brinalestrant in the active site of ERα [126]. Brinalestrant has been investigated in preclinical models. Brinalestrant has shown potent antitumor activity in breast cancer xenograft models with activating mutations in the ESR1, as well as in tamoxifen-sensitive and tamoxifen-resistant breast cancer xenografts [127,128]. A phase I study has investigated the effect of brilanestrant in advanced ER + breast cancer in postmenopausal women [128].

## 7. Concluding Remarks

Development of endocrine therapy resistance is a critical issue in the treatment of estrogen receptor-positive breast cancer in the adjuvant and metastatic setting that leads to treatment failure and breast cancer recurrence. In an effort to combat this hurdle, novel therapeutic approaches have been developed that either target estrogen receptor directly or modulate estrogen receptor signaling or modulate the cell cycle. Despite the advancements, the cornerstone of endocrine therapy for estrogen receptor-positive breast cancer remains SERMs, aromatase inhibitors, and oral SERDs. The mechanisms of development of acquired resistance of breast cancer cells against endocrine therapy have not been fully elucidated. Different mechanisms have been proposed, suggesting that the development of acquired resistance might be attributed to multiple mechanisms that could potentially act synergistically with each other, and more importantly, different mechanisms might be implicated in the development of resistance in each individual patient. Undoubtedly, further research is needed into the following directions: (i) mechanisms of acquired resistance to endocrine therapy; (ii) appropriate combinations of drug treatments for estrogen receptor positive breast cancer; (iii) appropriate sequence of treatments in advanced and metastatic estrogen receptor positive breast cancer. Special focus is needed on the role of ESR1 mutation, as it is the key driver of resistance in metastatic breast cancer. The recent FDA approval of the first PROTAC for advanced or metastatic ER-positive HER2-negative ESR1-mutated breast cancer is really encouraging and opens the road for the introduction of PROTACs in the pharmacotherapy of breast cancer as well as other diseases. The approval of the first PROTAC for the above indication highlights the high priority of developing novel drugs for endocrine therapy-resistant estrogen-positive breast cancer. A personalized approach to the clinical problem of acquired resistance to breast endocrine therapy would be more appropriate. Novel drugs that target estrogen receptor alpha that have been developed or are under development could potentially provide more therapeutic options. Future research efforts should focus on the identification of biomarkers that could enable early diagnosis of acquired resistance as well as personalized therapy guiding decisions on appropriate medication choice for the individual estrogen receptor-positive breast cancer patient. Molecular signatures could contribute to the identification of patients vulnerable to the acquisition of endocrine therapy resistance as well as to the identification of the putative mechanism.

## Figures and Tables

**Table 1:** Endocrine therapy for estrogen receptor positive breast cancer.

Types of Agents	Mechanism of Action	Indication or Putative Field of Research
LH-RH agonists	Reduction in the circulating concentrations of estrogen (ovarian suppression) in premenopausal women through their action on the hypothalamus–pituitary–ovarian axis	GnRH analogs are employed commonly in premenopausal women with estrogen receptor positive breast cancer in combination with estrogen antagonists or chemotherapy agents
Aromatase inhibitors	Inhibition of aromatase enzyme and reduction in estrogen levels	Adjuvant treatment of estrogen receptor positive breast cancer in postmenopausal women; chemoprevention for estrogen receptor positive breast cancer; metastatic estrogen receptor positive breast cancer
Selective estrogen receptor modulators (SERMs)	Inhibition of estrogen receptor through changes in estrogen receptor structure and cofactor recruitment.	Adjuvant treatment of estrogen receptor positive breast cancer in premenopausal and postmenopausal women; chemoprevention for estrogen receptor positive breast cancer; metastatic estrogen receptor positive breast cancer
Selective estrogen receptor degraders (SERDs)	Degradation of estrogen receptor	The SERD elacestrant has been approved for the treatment of postmenopausal women or adult men with ER-positive, HER2-negative, estrogen receptor 1 (ESR1)-mutated advanced or metastatic breast cancer with disease progression following ≥1 line of endocrine therapy
Complete Estrogen Receptor Antagonists (CERANs)	Blocking of both transcriptional activation domains of estrogen receptor.	Under investigation in patients with advanced or metastatic estrogen receptor positive breast cancer
selective estrogen receptor covalent antagonists (SERCAs)	Inactivation of both ER wild-type and mutated ER by covalently targeting a unique cysteine residue [cysteine-530 (C530)] that is not present in other hormone receptors.	Currently under evaluation in estrogen receptor-positive breast cancer patients under prior endocrine therapy

## Data Availability

No new data were created or analyzed in this study. Data sharing is not applicable to this article.
